# Effect of integrating traditional and modern healthcare systems on tuberculosis case detection in Ethiopia: a cluster randomized controlled study

**DOI:** 10.1186/s40249-024-01270-9

**Published:** 2025-03-03

**Authors:** Desalegne Amare, Kefyalew Addis Alene, Fentie Ambaw

**Affiliations:** 1 School of Health Sciences, College of Medicine and Health Sciences, Bahir Dar University, Bahir Dar, Ethiopia; 2 School of Population Health, Curtin University, Bentley, Perth, WA Australia; 3 Geospatial and Tuberculosis Research Team, Telethon Kids Institute, Bentley, Perth, WA Australia; 4 School of Public Health, College of Medicine and Health Sciences, Bahir Dar University, Bahir Dar, Ethiopia

**Keywords:** Tuberculosis, Case detection, Service integration, Traditional care, Modern healthcare system, Low case detection

## Abstract

**Background:**

Low tuberculosis (TB) case detection remains a major challenge in achieving the End TB targets. New strategies that consider local contexts are needed in countries with high TB burdens like Ethiopia. This study examined the effect of integrating traditional and modern TB care to increase the TB case detection rate.

**Methods:**

A cluster randomized controlled trial was conducted from February 2023 to January 2024 in six districts of South Gondar Zone, Northwest Ethiopia, where districts were randomly assigned to intervention or control groups. The interventions included training, screening, and referral of presumptive TB patients, delivered over one year, while the control group continued with the standard passive case detection approach. A paired *t*-test and two sample independent *t*-test were used to compare baseline and end line data for both groups. Cohen's *d* was also used to compare the effect size between the intervention and the control groups. A mixed-effect Poisson regression was employed to determine the association between the dependent variable and the exposure variables.

**Results:**

In the intervention group, a total of 620 TB cases were identified post-intervention, compared with 473 cases pre-intervention, including 14 cases identified through referrals by traditional care providers. In contrast, the control group identified 298 TB cases post-intervention and 279 pre-intervention. The TB detection rate increased to 93 cases per 100,000 population in the intervention group, making an approximate 29.2% improvement, compared to a 2.9% increase in the control group. Integrating traditional care with the modern healthcare system significantly increased case detection, with a standardized mean difference of 2.6 (95% confidence interval *CI*: (1.8, 3.5; *t* = 8.3; *P* < 0.001) in a two-sample independent *t*-test.

**Conclusions:**

Integrating traditional care with the modern healthcare system significantly increased TB case detection in high-burden settings. This approach not only enhances current TB control strategies but also has potential applications in managing other chronic diseases in resource-limited areas. Future research should evaluate the cost-effectiveness, scalability, and sustainability of this integrative model.

*Trial registration* Unique Protocol ID: 353/2021. ClinicalTrials.gov ID: NCT05236452. The date recruitment began: July 1, 2022. Registration date: July 22, 2022.

**Supplementary Information:**

The online version contains supplementary material available at 10.1186/s40249-024-01270-9.

## Background

TB remains a significant global public health concern, affecting an estimated 10.8 million people and resulting in 1.3 million deaths in 2023 [1]. Of these deaths, 90–95% were found in low- and middle-income countries (LMICs) [2, 3], and more than 87% of global TB cases were reported in 30 high-burden countries for TB [4]. Additionally, TB continues to be a significant public health problem in Africa, accounting for 23% of new cases and 31% of TB-related deaths [5]. The average TB incidence rate between 2010 and 2019 across 173 countries was 27.48%, with marked spatially stratified heterogeneity by country type and development stage, particularly in LMICs. This incidence of TB dramatically decreased in upper-middle-income countries compared with high-income countries, and the incidence of TB declined as the development stage increased [6].

The World Health Organization (WHO) estimated that globally, more than 4.1 million TB cases either go undiagnosed or are not reported to national TB programs [7]. Approximately, 60% of TB cases in LMICs remain undetected [8], with the majority occurring in Asian and African countries [9]. These undiagnosed cases continue to transmit of TB within the community [10], adversely affecting treatment outcomes [11].

Early case detection and treatment are crucial for improving TB control programs [12]. However, achieving the End TB targets remains challenging, especially in high-burden countries where the majority of missing cases are believed to be found [13]. Additionally, individuals with TB identified through passive case finding face a significant delay in diagnosis and treatment [14, 15]. In Ethiopia, the case detection rate (CDR) is low, even though the Health Sector Transformation Plan of Ethiopia sets a goal of achieving an 87% detection rate [16]. According to the 2021 national TB report, more than 29% of TB cases remain undiagnosed and untreated nationally [17], with this figure rising to 39% in the Amhara regional state [18]. Geographical disparities are evident, as seen in the Tigray region where the CDR dropped from 16% in 2016 to 7.73% in 2018 [19]. Meanwhile, the average case notification rates per 100,000 people were 126.4 and 131.4 in the Amhara and Oromia, respectively [20].

Several factors affect the TB case detection rate, with common challenges including lack of knowledge, resource shortages, inadequate health services, lack of a well-designed health system, poor coordination between sectors, lack of training and health services accessibility, lack of good referral linkage, low social support and lack of financial support [21–24]. Other significant barriers include staff shortages, limited transportation to healthcare facilities, inadequate medical infrastructure, and suboptimal laboratory services for the diagnosis of TB [25]. Many TB patients preferred to visit traditional care providers such as religious leaders and traditional healers before visiting hospitals and health centres [21–24]. This resulted in delays in the diagnosis and treatment of TB in resource-limited settings. The consequences of failure to early case detection and treatment increased the risk of death, severe illness, and transmission of TB in households and communities [26–30]. Previous studies showed that missed pulmonary TB cases can transmit infection to 10–15 people per year [31].

In the past, various interventions aimed at increasing case detection rates have been implemented. These interventions included training for health professionals, health extension workers, and volunteers [32–34], inmate peer education [35], and advocacy, communication, and social mobilization efforts [36]. Although these interventions increased case detection rates, they were not sustainable for several reasons. Firstly, the interventions often overlocked the cultural, social, and spiritual contexts critical for maintaining long-term outcomes [35]. Secondly, many previous interventions suffered from methodological flows and lacked significant community involvement [36]. Thirdly, the interventions did not take into consideration indigenous knowledge and practices essential to the communities they served, such as herbal medicine and faith-based care. For instance, in Sudan, 89.1% of patients use herbal medicine for healing [37], and in Ethiopia, up to 90% of the population seek healing through traditional healers and holy water [38].

Traditional healers have deep-rooted connections within their communities and often serve as the first point of contact for individuals seeking healthcare services. However, modern healthcare systems often overlook these practitioners, missing critical opportunities for TB case detection and collaboration. Neglecting these traditional healing sites can hinder the goal of ending TB. Therefore, integrating traditional and modern healthcare approaches is important for enhancing TB case detection in high-burden settings. This cluster randomized controlled trial aimed to determine the effect of integration on improving TB case detection rates in northwest Ethiopia.

## Methods

### Study setting

The study was conducted in the South Gondar Zone of the Amhara Regional State, in Northwest Ethiopia, which comprises 13 rural districts and eight town administrations. Each control district had an average population of 142,196, while the intervention district had an average population of 223,181. The study was conducted from February 2023 to January 2024. The average population of each cluster (catchment population of each health facility) was 27,403 people.

### Diagnostic and treatment of TB in the study setting

Diagnosis in this zone relies on identifying individuals who meet the clinical criteria for presumptive TB, followed by evaluation and confirmatory testing using sensitive methods. In both the control and intervention groups, TB was microbiologically confirmed using molecular World Health Organization rapid diagnosis (mWRD) tests, including the Xpert MTB/RIF, which is the standard for bacteriological confirmation in Ethiopia. All health facilities followed the national TB/DR-TB diagnostic and treatment algorithms. The lateral flow lipoarabinomannan assay (LF-LAM) was the preferred initial diagnostic test for people living with HIV. However, in cases where this test was not available on the same day, sputum microscopy tests were used to prevent diagnostic delays, and specimens were sent later for Xpert or other rapid diagnostic tests.

Patients diagnosed with bacteriologically confirmed TB undergo drug resistance screening for rifampicin using rapid drug susceptibility test (DST) techniques. Those with abnormal radiological findings on chest X-rays have sputum samples tested with mWRD. For patients who had rifampicin-resistant (RR)/multidrug-resistant (MDR)-TB, a second-line drug susceptibility test (SL-DST) was performed using a line probe assay (LPA) within one week of initiating the RR/MDR-TB treatment regimen. If resistance is detected via SL-LPA, further DST was conducted using phenotypic methods while treatment was managed based on the LPA result. All presumptive or confirmed TB patients were also tested for HIV. In cases where TB diagnosis remained uncertain despite negative bacteriological results, further investigations were considered [17].

### Trial registration

The trial was registered on ClinicalTrials.gov with the registration ID ClinicalTrials.gov: NCT05236452. Recruitment began on July 1, 2022, and the registration date was July 22, 2022. The trial also has a unique protocol ID of 353/2021. The ethical review report for this cluster randomized controlled trial is appended as additional file 1.

### Study design and randomization

**Fig. 1:**
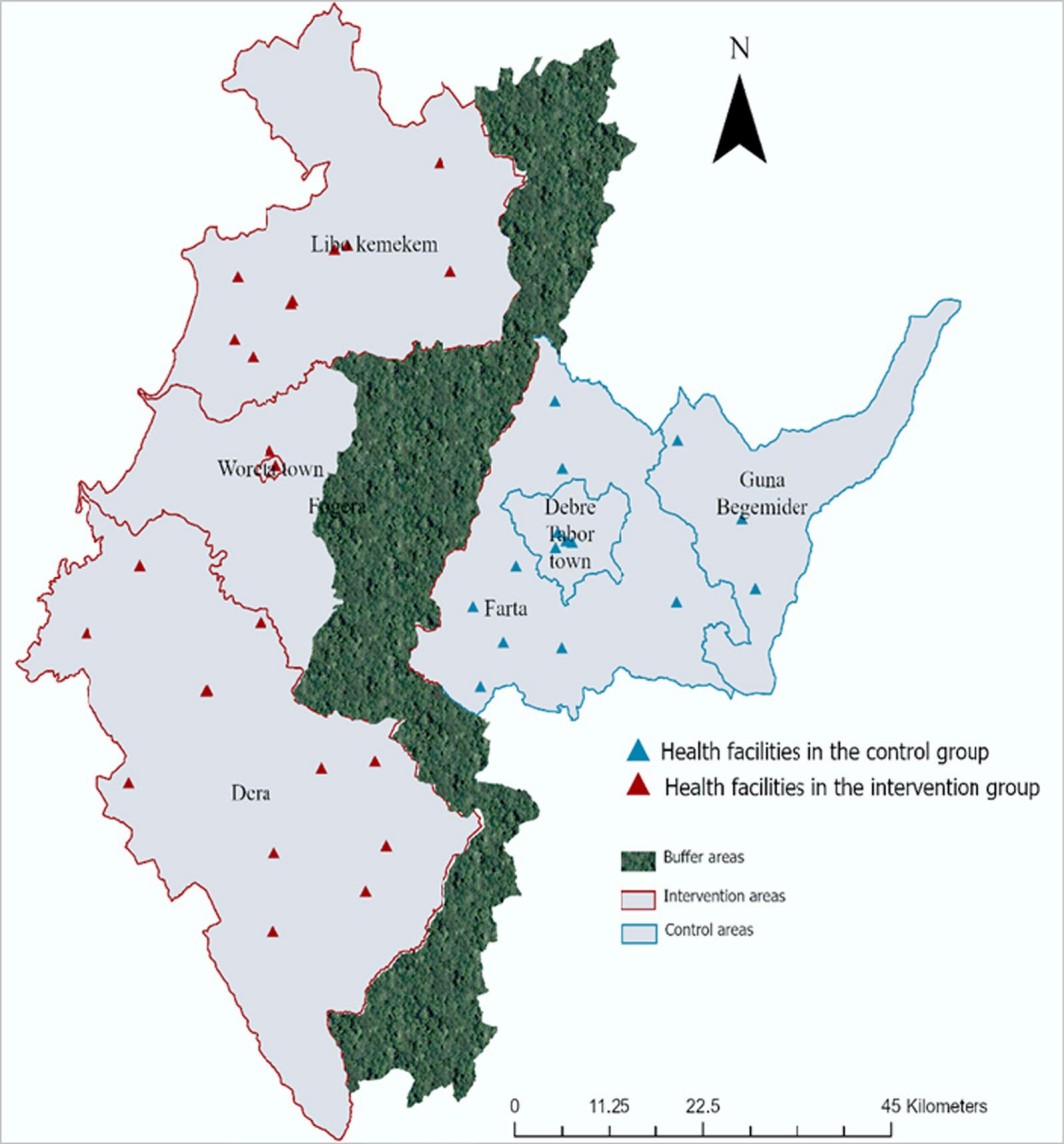
Map showing intervention and control areas’ boundaries, and location of health facilities

A cluster randomized controlled trial was conducted in the South Gondar Zone, Northwest Ethiopia. The zone comprises thirteen districts and eight town administrations, from which four districts and two town administrations were randomly selected using the random.org website. Two districts and one town administration were randomly assigned to the intervention group, and the remaining two districts and one town administration were assigned to the control group. All health facilities located in the selected districts and town administrations were included in the study. Forty health facilities within these selected areas were included in the study—24 in the intervention group and 16 in the control group. In order to avoid information contamination between the groups, it was arranged that there were districts that were not included in the study (Fig. 1).

All health facilities used similar TB screening and diagnostic approaches, and the distribution of supplies and reagents was uniform across all districts. Passive case detection was the standard approach. In the intervention areas, traditional care providers identified individuals with symptoms of TB and referred them to modern healthcare centres for diagnostic services. Additional information on randomization can be found in the protocol published elsewhere [39].

### Interventions

The intervention in this study involved integrating traditional healthcare with the modern healthcare system to increase TB case detection. Traditional care providers are defined as practitioners who provide informal patient care, including traditional healers and religious leaders. The intervention aimed to increase detection rates by screening patients and referring presumptive TB patients from traditional healers and holy water sites to health facilities.

The intervention was implemented in various phases. The first was the preliminary phase, where an exploratory qualitative study was conducted to assess the acceptability of integration published elsewhere [40]. The training manuals were developed separately for traditional and modern healthcare providers and then standardized through a workshop involving physicians, public health professionals, microbiologists, nurses, TB officers, and experts from non-governmental organizations.

The second phase involved providing training for both traditional and modern healthcare providers based on the standardized manuals. Training was provided in three rounds, emphasizing increasing participants’ knowledge, favorable attitudes, and skill acquisition. In the first round, five days of training were provided for traditional healthcare providers and two days of training for modern healthcare providers. The second and third rounds of training were provided one day each in the third and sixth months following the initial training. The training was conducted by principal investigators and TB experts who had received training of trainers (TOT) in the national TB treatment and control program. Furthermore, the training content aimed to encourage healthcare providers to have a positive attitude toward traditional healers and religious leaders and promote cooperation between them. Religious leaders, traditional healers, and healthcare providers all believed in and respected religion. The other aim was to bring about behavioral change among healthcare providers regarding the acceptance of traditional healing and holy water spiritual healing.

The third phase involved traditional healers and religious leaders screening all patients and referring presumptive cases to nearby health facilities using standardized tools. The screening was conducted based on history, inspection of the patient's general appearance, and palpation to identify lymphadenopathy. The details of screening and referral formats are indicated in the supplementary materials (S1-Table 1 and S2-Table 2).

The fourth phase involved an end line outcome assessment that was conducted at the end of one year of intervention. Details of the operational procedures for the intervention packages are provided in a published protocol [39] and available in the supplementary materials (S3-Table 3 and S4- Fig. 1).

### Control

In the control group, the standard passive case-finding strategy was continued. This approach relies on symptomatic individuals self-presenting at health facilities for diagnostic testing. To assess the effectiveness of the intervention, we compared the TB case detection rates between the intervention and control groups. The findings from the control group served as a baseline to evaluate the differential impact of integrating traditional care practices in the intervention group.

### Outcome measurement and definition

Baseline case detection was assessed to determine if a significant difference was observed between the control and intervention groups. The TB case detection rate was calculated by dividing the total number of TB cases for the year by the mid-year total population of the catchment area and, then multiplying by 100,000 to determine the estimated cases per year. The mid-year total population was computed by adding the population figures for the 1st and 12th months of the same year and dividing the sum by two. In addition, the difference between the baseline and end line following the one-year intervention was determined by subtracting the number of notified TB cases during the intervention year from the baseline data collected one year before the start of the intervention. This measurement approach provided a standardized method for evaluating the effectiveness of the intervention in detecting TB cases within the study population.

### Data extraction and procedure

**Fig. 2:**
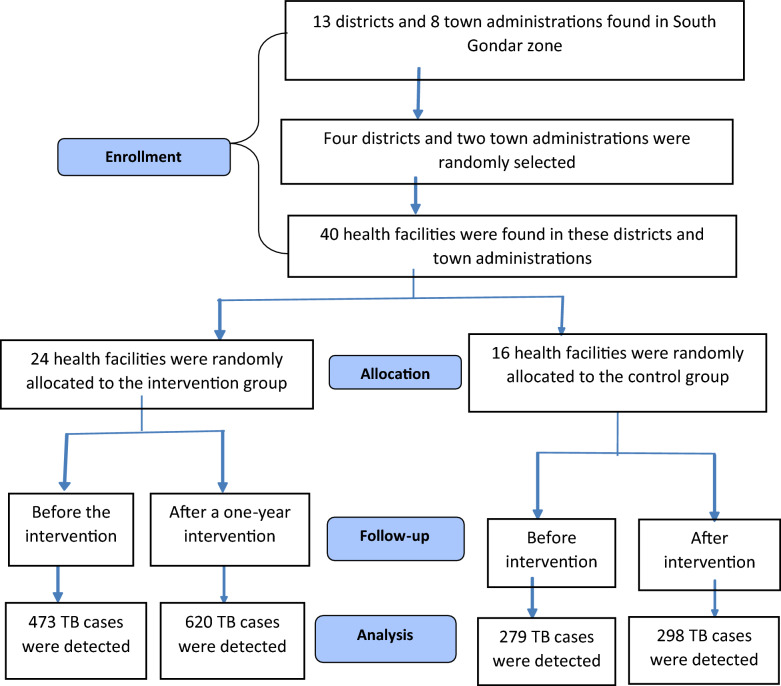
Trial profile of study participants according to the criteria recommended in the CONSORT guideline. *CONSORT* Consolidated Standards of Reporting Trials

The data were extracted from TB registration books and the District Health Information Software (DHIS2) database, which was developed by a global collaboration managed by the HISP Centre at the University of Oslo. The data were extracted by trained public health and health informatics experts. The techniques and procedures are illustrated in Fig. 2.

### Data quality assurance and safety monitoring

Data quality was ensured through adequate training, regular supervision, and blinding of outcome assessors regarding intervention allocation. The data were taken from the DHIS2 database, which is managed by zonal, regional, and national health systems. Methodological components were ensured for quality by following the Consolidated Standards of Reporting Trials (CONSORT) 2010 statement extension to cluster randomized controlled trial study guidelines [41]. Additionally, the Template for Intervention Description and Replication (TIDieR) checklist was used to enhance the quality of our reports [42]. Trained and experienced TB experts evaluated the outcomes of the intervention. The data were analyzed using intention-to-treat (ITT) principles [43]. Throughout the trial period, no safety concerns were identified despite thorough monitoring. Further details are provided in the supplementary material (S5-Table 4).

### Data management and analysis

The data was compiled using Excel spreadsheets and then exported to Stata software version 17 (StataCorp. 2021. Stata: Release 17. Statistical Software. College Station, TX: StataCorp LLC, Texas, USA) for analysis. The total number of patients screened and referred by each traditional healer and religious leader during the study period was determined from their meticulously kept records. These records were cross-checked with TB registration data at health facilities and validated against entries in the DHIS2 database to ensure accuracy and consistency.

Descriptive statistics were used to summarize the baseline characteristics. Chi-square tests were employed to compare the characteristics of both the control and intervention groups at baseline and end line. Since the data  did not adhere to a normal distribution, as evidenced by the histogram, data transformations were applied to achieve normality. Among the transformations tested (logarithmic, square root, and inverse), the logarithmic transformation resulted in a normal distribution. After conducting statistical analyses on the transformed data, the results were back transformed using exponentiation.

Paired *t*-tests compared pre- and post-intervention measurements within groups, while two-sample independent *t*-tests compared differences between the intervention and control groups. Effect sizes were calculated using Cohen's d to quantify the magnitude of differences, computed as d = (M₂ − M₁) ⁄ SDₚₒₒₗₑd, where M₁ and M₂ are the means of the baseline and end-line measurements, respectively, and SDₚₒₒₗₑd is the pooled standard deviation.

To assess the effect of the intervention on TB detection rates, we employed a multilevel mixed-effects Poisson regression model suitable for counting data and accounting for clustering at the district level. The intra-cluster correlation coefficient was calculated according to the following formula: ICC = $\frac{{\sigma u}^{2}}{{{\sigma u}^{2}+\sigma e}^{2}}$. Model fit was evaluated using the Akaike Information Criterion (AIC) and Bayesian Information Criterion (BIC), with the lowest values indicating the best-fitting model. Statistical significance was determined at a *P*-value of less than 0.05.

## Results

### Study characteristics

In the intervention group, a total of 620 TB cases were identified post-intervention compared to 473 pre-intervention. Throughout the one-year study period, an average of 1080 individuals sought care at each traditional care setting. Among these, 260 presumptive TB cases identified by traditional care providers were referred to nearby health facilities for confirmation of TB diagnosis. Out of the cases referred, 14 patients confirmed they had active TB (5 pulmonary TB and 9 extrapulmonary TB), while 171 clients were found to be free of TB, and 75 were lost to follow-up. TB diagnosis was conducted using both bacteriological and clinical methods. In the control group, there were 298 TB cases post-intervention and 279 pre-intervention.

Table 1 presents the baseline characteristics of socio-demographic variables in both the intervention and control groups. Bacteriologically confirmed pulmonary TB cases accounted for 27% in the intervention group and 22% in the control group, while extrapulmonary TB accounted for 47% in the intervention group and 44% in the control group.

**Table 1:** Baseline characteristics of study participants in the control and intervention groups in south Gondar zone, northwest Ethiopia

Variables		Baseline			End-line		
		Control group (*N* = 279)	Intervention group (*N* = 473)	*P*-value	Control group (*N* = 298)	Intervention group (*N* = 620)	*P*-value
Sex	Male	150	232	0.959	167	314	0.483
	Female	129	241	0.650	131	306	0.258
Age in years	0–4	1	6	0.197	5	14	0.290
	5–9	6	10	0.850	3	19	0.037
	10–14	16	24	0.995	4	24	0.014
	15–19	43	58	0.746	15	34	0.363
	20–24	49	70	0.850	47	54	0.540
	25–34	57	122	0.513	70	146	0.527
	35–44	38	83	0.352	53	118	0.286
	45–54	34	45	0.518	38	98	0.144
	55–64	24	24	0.316	27	60	0.183
	≥ 65	17	31	0.699	36	53	0.956
Types of TB	Smear positive PTB	47	103	0.476	67	164	0.192
	Smear negative PTB	82	82	0.273	95	145	0.982
	EPTB	146	269	0.606	131	292	0.278
	Relapse TB	4	19	0.456	5	19	0.258

*TB* Tuberculosis, *PTB* Pulmonary tuberculosis, *EPTB* Extrapulmonary tuberculosis

At baseline, there were no significant differences between the groups regarding sex, age distribution, or types of TB (*P* > 0.05). At the end-line, significant differences were observed in the age groups 5–9 years (*P* = 0.037) and 10–14 years (*P* = 0.014), with higher case numbers in the intervention group. At the end line, the intervention group showed a significant increase in TB cases among children aged 5–9 years and 10–14 years compared to the control group. No significant differences were observed in other age groups or the distribution of TB types between the groups (Table 1).

### Effect of the intervention on TB case detection rate

**Fig. 3:**
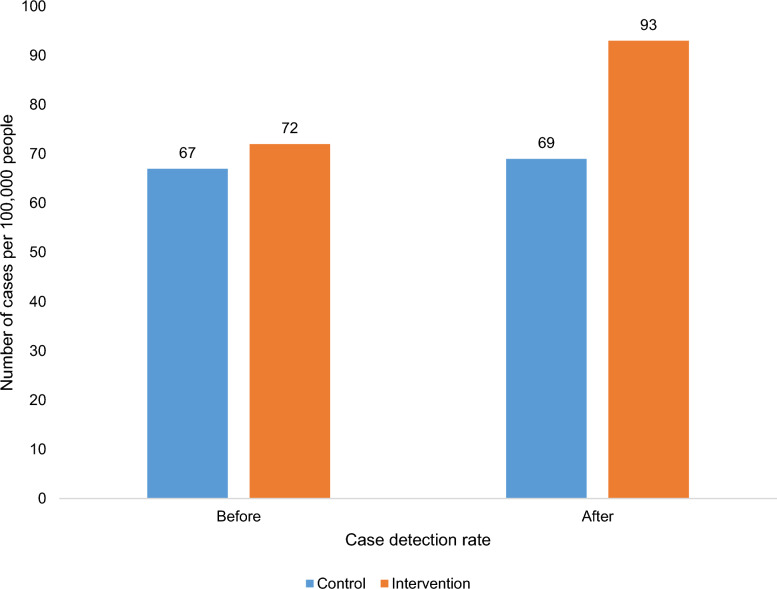
Comparison of tuberculosis case detection rate between the intervention and control groups

After the implementation of the intervention, the TB detection rate in the intervention group increased by 21 cases per 100,000 population, rising from 72 to 93 cases per 100,000. In contrast, the control group showed a minimal increase of 2 cases per 100,000 population, from 67 to 69 cases per 100,000 (Fig. 3).

A two-sample independent *t-*test comparing post-intervention data revealed that the integration of traditional care with the modern healthcare system significantly increased the TB case detection rate. The standardized mean difference between the intervention and control groups was 2.621 (95% *CI*: 1.8, 3.5; *t* = 8.3; *P* < 0.001) (Table 2).

**Table 2:** A two-sample independent *t*-test was used to compare the mean annual TB cases at baseline and end-line measurements between the control and intervention groups, in northwest Ethiopia

Variables	Control group (*N* = 16)		Intervention group (*N* = 24)		Standardized group mean difference	*t*-test	*P-*value
	Mean (± *SD*)	*se*	Mean (± *SD*)	*se*			
Before	11.1(3.0)	0.8	11.2(3.1)	0.6	0.1	0.2	0.559
After	11.4(2.8)	0.7	18.1(2.3)	0.5	2.6	8.3	0.001

*SD* Standard deviation

Within the intervention group, a paired *t*-test comparing baseline and end-line data showed a significant increase in case detection, with a standardized mean difference of 1.8 (*t* = 6.4; *P* < 0.001). No significant change was observed in the control group over the same period (Table 3).

**Table 3:** A paired *t*-test comparing baseline and end-line mean annual TB cases between the control and intervention groups in northwest Ethiopia

Variables	Control group (*N* = 16)				Intervention group (*N* = 24)			
	Mean (± *SD*)	*se*	Standardized group mean difference	*t*-test (*P*-value)	Mean (± *SD*)	*se*	Standardized group mean difference	*t*-test (*P*-value)
Before	11.4 (3.1)	0.8	− 0.004	− 0.01 (0.495)	13.9 (2.4)	0.5	1.8	6.4 (0.001)
After	11.4 (2.8)	0.7			18.2 (2.2)	0.4		

*SD* Standard deviation

This study showed that the intervention resulted in a 29.2% increase in the detection rate within the intervention group, compared to a 2.9% increase in the control group. This study demonstrates that the implementation of the integration effectively increased the detection rate. The difference-in-difference was 19 (Table 4).

**Table 4:** Effect size estimation and difference in difference of TB case detection in pre- and post-treatment between the intervention and control groups in northwest Ethiopia

Variables	Control group		Intervention group	
	Before	After	Before	After
All forms of TB	279	294	473	620
TB cases per 100,000 population	67	69	72	93
Percentage change	2.9%		29.2%	
Difference in difference (DID)	19			

DID = (Intervention group post – Intervention group pre)–(Control group post–Control group pre). *TB* Tuberculosis

**Fig. 4:**
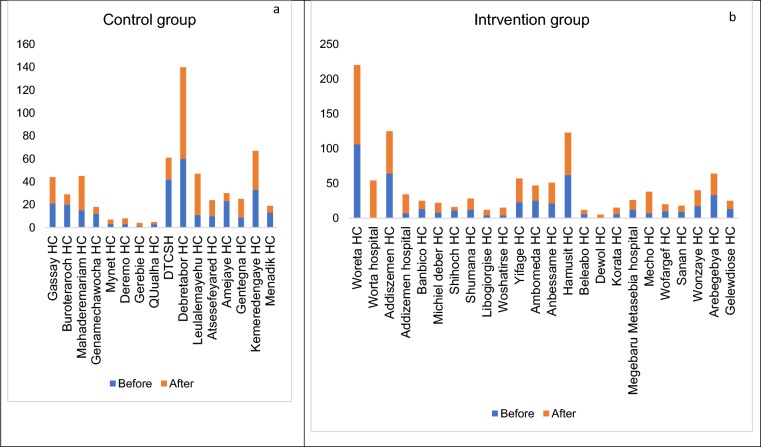
Tuberculosis case detection before-and after-intervention between the control and intervention groups in each health facility. **a**: Case detection before and after in control group in each facility. **b**: Case detection before and after in intervention group in each facility. *DTCSH* Debre Tabor comprehensive specialized hospital, *HC* Health center

TB case detection among health facilities in the control group did not change before or after the intervention. Some facilities experienced slight increases, while others saw decreases. In contrast, the intervention group demonstrated an increase in TB detection rates following the implementation of the intervention in facilities (Fig. 4).

### Multilevel mixed-effect Poisson regression

The multilevel mixed-effects Poisson regression model, accounting for clustering at the district level, demonstrated that implementing the intervention significantly increased TB case detection rates, with an incidence rate ratio (IRR) of 1.2 (95% *CI*: 1.1, 1.4). This indicates a 22.3% higher detection rate in the intervention group compared to the control group after adjusting for other factors. Age was a significant factor, with older age groups exhibiting higher detection rates. For example, individuals aged 55 and above had an IRR of 1.4 (95% *CI*: 1.2, 1.5), suggesting increased detection in the older age group.

The inclusion of random effects at the district level improved the model fit, capturing unobserved heterogeneity between districts. The reduced variance in the full model indicates that accounting for the intervention and other covariates explained much of the variability across districts (Table 5).

**Table 5:** Mixed effect Poisson regression to estimate the effect of the intervention on case detection in Ethiopia

Variables	Null model		Model 1		Model 2		Model 3 (Full model)	
	IRR (*se*)	95% *CI*	IRR (*se*)	95% *CI*	IRR (*se*)	95% *CI*	IRR (*se*)	95% *CI*
Intercept	14.4 (2)	10.9, 18.9	5.9 (0.7)	4.6, 7.5	14.6 (3.2)	9.4, 22.5	5.7 (0.7)	4.5, 7.1
Intervention					1.3(0.1)	1.2, 1.5	1.2 (0.1)	1.1,1.4
Male			1.3 (0.2)	0.9, 1.8			1.2 (0.2)	0.8, 1.7
Female			1.2 (0.2)	0.8, 1.7			1.1 (0.2)	0.8, 1.6
Age in year								
0–14			1.1 (0.1)	0.9, 1.3			1.1 (0.1)	0.9, 1.3
15–24			1.2 (0.1)	1.1, 1.3			1.2 (0.1)	1.1, 1.3
25–34			1.2 (0.1)	1.1, 1.4			1.2 (0.1)	1.1, 1.4
35–44			1.2 (0.1)	1.1, 1.4			1.2 (0.1)	1.1, 1.4
45–54			1.3 (0.1)	1.1, 1.6			1.3 (0.1)	1.1, 1.5
≥ 55			1.4 (0.1)	1.2, 1.6			1.4 (0.1)	1.2, 1.5
Types TB								
PTB positive			0.7 (0.1)	0.5, 0.9			0.7 (0.1)	0.5, 1.1
PTB negative			0.7 (0.1)	0.5, 0.9			0.7 (0.1)	0.5, 1.1
EPTB			0.7 (0.1)	0.5, 0.9			0.7 (0.1)	0.5, 1.1
Relapse TB			0.8 (0.2)	0.5, 1.1			0.8 (0.2)	0.6, 1.2
Random effects								
Variance(σ2)	0.7 (0.2)		0.1 (0.1)		0.6 (0.2)		0.1 (0.1)	
ICC(ρ)	0.9		0.5		0.9		0.5	
AIC	698.4		609.4		679.1		600.5	
BIC	703.1		642.7		688.7		636.2	

*IRR* Incident rate ratio; *CI* Confidence interval; *TB* Tuberculosis; *EPTB* Extrapulmonary tuberculosis; *PTB* Pulmonary tuberculosis; *ICC* Intraclass correlation coefficient; *AIC* Akaike information criterion; *BIC* Bayesian information criterion

## Discussion

This cluster-randomized controlled trial aimed to integrate traditional healthcare services into TB control programs as a strategy to improve TB case detection. The interventions, which included training traditional healers and religious leaders to screen their clients for TB, refer presumptive TB cases to health facilities, and link them to TB clinics, resulted in a significant increase in TB case detection rates in northwest Ethiopia. This strategy shows promise for large-scale implementation across Ethiopia and sub-Saharan Africa.

The study revealed that integrating traditional care with the modern healthcare services significantly increased TB case detection in the intervention group compared to the control group. There was a 29.2% increase in TB case detection rates following the implementation of the intervention, which was higher than the 2.9% rate reported in the control group. This suggests that leveraging existing community resources, such as traditional healers and religious leaders, can complement formal healthcare systems in enhancing TB diagnosis and outcomes. Our findings align with previous studies that emphasize the significance of community engagement and decentralized approaches in TB control efforts [44–46].

Our study showed that individuals aged 15 years and older had a significantly higher rate of TB compared to those under 15 years old. Diagnosing TB in children is challenging due to nonspecific signs and symptoms, limited specimen material, and lower bacterial loads in specimens compared to adolescents and adults [47]. The prevalence of EPTB in the study area is notably high. The reasons for the high prevalence of EPTB are not well understood and need further research. In many parts of Ethiopia, nearly one-third of all TB cases are EPTB [47, 48]. Diagnosing EPTB involves multiple diagnostic methods such as histopathology, Xpert testing, and echocardiography, in addition to clinical findings consistent with EPTB. Often, EPTB is not confirmed microbiologically, leading to the initiation of anti-TB therapy based on clinical diagnosis [47, 49–51]. The high rates of EPTB in this setting may be influenced by several factors, including potential delays in diagnosis, limited access to healthcare facilities, and possible variations in host immunity or co-existing health conditions that predispose individuals to EPTB. Additionally, diagnostic practices and preferences, along with the high prevalence of certain risk factors in the region, could contribute to these elevated rates. Further research may be required to fully understand the underlying causes specific to this setting.

Other factors may contribute to the observed differences in TB detection rates between the intervention and control groups. Firstly, the training provided to traditional care providers likely enhanced their knowledge and skills in identifying TB symptoms and referring presumptive cases to healthcare facilities. Additionally, the establishment of referral linkages facilitated collaboration between traditional and modern healthcare systems, ensuring timely diagnosis and treatment initiation for TB patients [40]. Moreover, the culturally sensitive approach adapted in the intervention may have fostered greater trust and acceptance within the community, encouraging individuals to seek care for TB-related symptoms.

The study uses a methodologically strong design that can be trusted and utilized by policymakers, programmers and various partners. Although the intervention was successful in detecting TB in the community, several limitations should be considered. Some presumptive TB cases identified through the traditional care system lacked contact information, making it difficult to locate patients who did not reach the modern healthcare system. Additionally, patients may not communicate or present the referral letter to healthcare providers because they feel ashamed of having sought care from traditional healers. The cost-effectiveness of the intervention has not been studied and requires future investigation. Sustaining the intervention beyond the study period could pose significant challenges, highlighting the necessity for long-term commitment and support from relevant stakeholders. Long-term collaboration between the two systems may be challenging unless policymakers and stakeholders demonstrate interest in scalability, reproducibility, and continuity across all levels of healthcare facilities [52, 53].

Moving forward, efforts should be directed toward expanding the intervention to other high-burden TB settings and assessing its scalability and sustainability in real-world settings to enhance TB control and prevention. This is crucial for reducing TB transmission, promoting early diagnosis and treatment, and improving the social well-being of individuals affected by TB and their families. Furthermore, continuous monitoring and evaluation are essential to track long-term outcomes and address emerging challenges. Collaborative partnerships between government health agencies, non-governmental organizations, and community stakeholders are key to ensuring the successful integration of traditional care providers into the modern healthcare system.

## Conclusions

Implementing the intervention significantly increased TB case detection. Integrating traditional care with the modern healthcare system is a promising strategy to improve case detection in high-burden TB settings. The findings of this study showed the significance of engaging traditional healers and religious leaders as well as implementing decentralized approaches to achieve End TB targets. Relying solely on biomedical care strategies for TB control programs may not be beneficial for societies with traditional multicultural healing practices. By leveraging the expertise and reach of traditional healthcare providers, TB surveillance and diagnosis can be enhanced, ultimately contributing to the global initiative to eliminate TB as a public health concern. This intervention model may also work to identify and manage other chronic illnesses in resource-poor settings; therefore, further interventional studies should be conducted on cost-effectiveness, scalability at wider ranges, and sustainability of integration. Case detection rate significantly increased among adult patients compared to paediatrics patients.

## Supplementary Information

Additional file 1.

## Data Availability

The study datasets are available from the corresponding author and can be shared upon reasonable request.

## Abbreviations

CDR: Case detection rate
*CI*: Confidence interval
DOT: Direct observation therapy
EPTB: Extrapulmonary tuberculosis
HIV: Human immune deficiency virus
IRR: Incident rate ratio
LF-LAM: Lateral Flow Lipoarabinomannan Assay
LMICs: Low- and middle- income countries
LPA: Line-Proven Assay
MDR-TB: Multidrug resistance TB
mWRD: Molecular World Health Organization Rapid Diagnosis
PTB: Pulmonary tuberculosis
RR: Rifampicin resistance
SL-DST: Second-Line Drug-Sensitive Test
TB: Tuberculosis
WHO: World Health Organization
