# Supplementary material for: Effect of integrating traditional and modern healthcare systems on tuberculosis case detection in Ethiopia: a cluster randomized controlled study
Source: Infect Dis Poverty. 2025 Mar 3;14:16. doi: 10.1186/s40249-024-01270-9 (PMC11874796; doi:10.1186/s40249-024-01270-9)
Supplement: Supplementary file 1 — Additional file 1. [file 40249_2024_1270_MOESM1_ESM.docx]

**Appendix**

Annex 1: TB screening form used to detect TB presumptive by traditional practitioners

Name of the patient: __________________________

Date of visiting ________________ Address _________________town___________Telephone Number___________________

Screening date: _____________________________

| Signs and symptoms | Yes | No |
| --- | --- | --- |
| Any cough for two weeks more than two weeks |  |  |
| Persistent fever |  |  |
| Night sweating |  |  |
| Unintentional weight loss (if children, failure to weight gain, failure to thrive and malnutrition) |  |  |
| History of contact |  |  |
| History of previous TB treatment |  |  |
| Chest pain |  |  |
| Persistent shortness of breath (Dyspnea? |  |  |
| Weakness or fatigue |  |  |
| No appetite |  |  |
| Chills |  |  |
| Hemoptysis |  |  |
| Lymph node enlargement (lymphadenopathy) |  |  |
| Have you had contact with anyone having a cough or tuberculosis disease in the past year? |  |  |
| Do you have a medical condition, or are you taking medications? |  |  |
| Have you had contact with a family member or partner who has been diagnosed with TB/having coughed? |  |  |

If any of the above answers are "yes", instruct the client to wear the mask and refer for further evaluation to the nearby health institutions.

Commentes:_________________________________________________________________________________________________________________________________________________________________________________________________________________________________________________________________

Referral form used to screen presumptive TB cases by traditional care practitioners

Name of presumptive TB cases: ____________________________________________

Date of visiting ________________ Address _________________town___________

Telephone number if (available): ___________________

Screening date: ________________________________

Presenting signs and symptoms:

1._______________________2. ______________________3.______________________

4._______________________5.______________________6._______________________

Duration of symptoms for: _______________________________days/weeks/months/years

Date of refer _______________________________

Referral code: _______________________________

Refer from (Name of visiting traditional center)______________to _______________ health center/hospital

Comments:____________________________________________________________________

Name of the evaluator: _____________________________ Signature: _________________

**Annex 2: Intervention packages**

**Phase 1: Preliminary phase or training material preparation**

All interventional packages, including training materials and checklists, were developed in this phase. Experts were invited to critique training manuals, screening and referral formats, and fidelity checklists. Physicians, public health experts, language professionals, TB focal personnel, and traditional care providers were invited to critique the contents, depth, readability, and understandability of the training manuals, formats, and checklists. To further enhance, a two-day workshop was conducted and invited experts provided their insights. The training materials were comprehensive and covered knowledge, attitude, and skill domains. A manual prepared for traditional care providers included contents of causes, signs and symptoms, mode of transmission, patient screening and referring, diagnosis approach, case detection strategies, ways of improving treatment outcomes, the advantages of early detection and treatment, complications of late diagnosis and treatment, and control and prevention mechanisms of TB.

The training manual for healthcare providers also includes an overview of global and national TB epidemiology, community TB case detection, the importance of community engagement and service integration, the impact of integrating traditional and modern care, experiences of integration in different countries, strategies for integration, holistic patient care, patient-centered care, social and spiritual support for TB patients, and spiritual care. Finally, senior experts approved training manuals and checklists.

**Phase 2: Training provision**

Training was provided to both traditional and modern care practitioners in three rounds. During the first round, traditional practitioners (traditional healers and religious leaders) received 5 days of training, while healthcare providers (TB focal persons) were trained for two days. One-day trainings were conducted in the second and third rounds in the third and sixth months after the initial training session. The training sessions were facilitated by researchers and certified training trainers from the regional health bureau. Participants who scored 75% or higher on the post-test in the knowledge and attitude domains, and 100% in the skill domains, were recruited as implementers. Before starting the actual intervention, baseline data was collected and analyzed.

**Phase 3: Screening and referral of TB‑suspected cases**

A full-blown of the intervention was implemented during this phase. Traditional practitioners used standardized screening tools for screening patients suspected of having TB. Any suspected TB cases at traditional healer centers and holy water were referred to nearby health facilities in the intervention districts. Again, trained TB focal persons were screened patients referred from traditional practitioners and diagnosed based on the national TB treatment guideline. Progress evaluation of the intervention was assessed quarterly. Assigned experts performed regular monthly supervision. Trained assessors conducted intermediate outcomes assessment in the third and sixth months of the intervention. The post-test results of each participant were assessed and the trainees who scored the required mark were deemed capable of screening and referring patients using screening and referral formats (annex_2 TB screening and referral form).

**Phase 4: The end‑line evaluation**

The end-line outcome was assessed after the one-year duration of the intervention. The comparison was done between the end-line and baseline outcome (case detection rate) between the intervention and control group.

Table 1: Intervention packages, methods frequency, duration, dosage, and components of the intervention

| Activities | Methods | Frequency, duration, and doses | Contents of the training manuals |
| --- | --- | --- | --- |
| Training for both categories of practitioners (traditional and modern care providers) | Group-based training was provided by researchers | - The training was provided three times - 1^st^ round for five days - 2^nd^ round for 1 day at 3^rd^ months - 3^rd^ round for 1 day at 6^th^ months | - Training manual for traditional care providers includes the following contents: - To increase the knowledge of traditional care providers the following contents were included: - Definition of TB, - Causes, - Sign and symptoms, - Mode of transmission, - Prevention and control, - Advantage of early TB diagnosis and treatment, - Health and economic impact of TB on the families and the community, and - Integration of traditional and modern healthcare systems. - To create a favorable attitude of traditional care providers the following contents were included: - Healthcare providers’ cooperativeness towards integration, - Healthcare providers attitude towards traditional healers and religious leaders, - Positive perspective of healthcare providers towards herbal medicine and holy water, - Healthcare providers believe and respect religious leaders and traditional healers. - To acquire skills on screening and referring patients, we included contents of medical history taking, inspection of the overall general appearance of the patient and ability to identify lymphadenopathy through palpation. - **Training manual for modern care providers**   To increase the knowledge of modern healthcare providers the following contents were included:   - Global and national burden of TB, - Evidence for low case detection rate - Challenges to increase case detection rate, - The role of traditional care in increasing case notification and integration of modern healthcare with the traditional healthcare system to increase accessibility of alternative healing facilities and patient centred care were included. - The training was implemented through dictating, group discussion, case scenario and simulative exercise, video and role play to increase the skill of practitioners. |
| TB screening | - Subjective and objective data were gathered - Role-play sessions were performed | Screen patients every case for every day through one year | - To crate favorable attitude of healthcare providers the following contents were included: - Acceptance of providing drugs and holy water simultaneously by religious leaders - Cooperativeness of traditional care providers with healthcare providers - Healthcare providers believe and respect religious leaders and traditional healers |
| Screening patients who need spiritual support by healthcare providers | Group-based training and demonstration and role-play of how healthcare providers screen patients who need spiritual support | Screen patients every case for every day through one year | Use any signs and symptoms of stress, such as sadness, frustration, or emotional instability, as an indicator. |
| Referral linkage | Group-based training and individual-based practical/demonstration and role-play sessions were conducted on how to refer suspected patients | Refer patients every case for every day through one year | - Patient identification - Coughing for two weeks or   at least two sign and  symptoms of TB infection |

- Trainings were provided for traditional and modern healthcare practitioners
- Relevant contents were included in the manuals to acquire knowledge, skills and to create favorable attitude.
- Three rounds of trainings were provided for practitioners
- Pre and post tests were conducted to recruit the right implementers

Screening patients who came to the traditional care providers’ center

- Systematic screening of patients who had signs and symptoms of TB
- Systematic screening was done by taking medical history, inspection, and palpation of lymphadenopathy as well as using both subjective and objective data.

Apply referral linkage

Intervention

Sup 4_figure 1: The theory shows the intervention process and outcomes

Intermediate outcomes

- Traditional practitioners became skilled in screening and referring presumptive cases to modern healthcare system
- Able to refer patients from modern healthcare system to traditional practitioners if necessary
- Acquired knowledge and skills on TB screenings and referral linkage/integration
- Brought behavioral change
- Minimize stigma and discriminations of traditional care providers
- Increase awareness on the role of traditional practitioners

End-line outcome

- Willing to participate in the training
- Complete the trainings
- Use the screening and referral forms properly
- Willing to monitor and evaluate the progress of the integration
- Score the minimum pass mark
- Apply the training manual as prescribed
- Cooperate to screen and refer presumptive cases
- Acquired necessary skills and knowledge that capable to identify cases

Assumptions

Increase TB case detection rate

**Annex 3**

Table 2: The TIDieR (Template for Intervention Description and Replication) Checklist

| Item | Primary paper (page or appendix number) | Other (details) |
| --- | --- | --- |
| Name or describe the intervention | Impact of integrating traditional care with modern health care on TB detection |  |
| Describe the rationale, theory, or goal of the elements essential to the intervention. | TB case detection rate remains a global challenge, particularly, since this is a major public health concern in low- and middle-income countries. Collaboration between traditional healers and healthcare workers would lead to increased TB case detection, particularly among underserved populations in high TB burden settings. Therefore, aimed to investigate the effectiveness of integrating traditional care with modern healthcare on the TB case detection rate in Ethiopia. |  |
| Materials: Describe any physical or informational materials used in the intervention, including those provided to participants or used in intervention delivery or the training of intervention providers. Provide information on where the materials can be accessed | TB screening and referral formats were used in the intervention. The published protocol was used as guidance to implement the intervention. These materials can be accessed in the appendix section of this paper and find the protocol at BMC trials: <https://doi.org/10.1186/s13063-023-07559-8> |  |
| Procedures: Describe each of the procedures, activities, and/or processes used in the intervention | All interventional packages, training manuals, checklists, and formats were developed and standardized. Training sessions were provided to both health professionals and traditional care providers in three rounds. The referral linkage model facilitated the identification and referral of TB cases from traditional care centers to modern healthcare facilities, thereby enhancing TB case detection. An outcome assessment was conducted to comparison between the end-line and baseline outcome (case detection rate) between the intervention and control groups. |  |
| For each category of intervention provider, describe their expertise, background, and any specific training given | The intervention providers were trained traditional and modern care practitioners. Separate training sessions were prepared for various lengths of days in three rounds. Pre-and post-tests were provided for both groups and those who scored pass marks proposed to implement the intervention. |  |
| Describe the modes of delivery (e.g. face-to-face or by some other mechanism, such as internet or telephone) of the intervention and whether it was provided individually or in a group | The mode of delivery was face-to-face |  |
| Describe the type(s) of location(s) where the intervention occurred, including any necessary infrastructure or relevant features | The intervention was provided at the cluster or facility level |  |
| Describe the number of times the intervention was delivered and over what period including the number of sessions, their schedule, and their duration, intensity, or dose | TB screening and referring of suspected cases were conducted for one year. |  |
| If the intervention was planned to be personalized, titrated, or adapted, then describe what, why, when, and how | The interventions (training manuals, formats, and checklists) were developed by researchers and standardized by conducting workshops. Senior TB experts were invited to comment on it. Finally, researchers approved the document. |  |
| If the intervention was modified during the study, describe the changes (what, why, when, and how). | The intervention was undergone based on the predefined plan. |  |
| Planned: If intervention adherence or fidelity was assessed, describe how and by whom, and if any strategies were used to maintain or improve fidelity, describe them | A fidelity checklist was used to monitor and evaluate the intervention activities that are amenable to measurement: adherence, dosage, quality of intervention delivery, participant responsiveness, and program differentiation. |  |
| Actual: If intervention adherence or fidelity was assessed, describe the extent to which the intervention was delivered as planned | The implementation of the intervention was to stick to the published protocol and fidelity checklist. |  |
